# Supplementary material for: Identification of photoperiod-induced specific miRNAs in the adrenal glands of Sunite sheep (Ovis aries)
Source: Front Vet Sci. 2022 Jul 22;9:888207. doi: 10.3389/fvets.2022.888207 (PMC9354845; doi:10.3389/fvets.2022.888207)
Supplement: Supplementary file 1 [file Data_Sheet_1.ZIP › Supplementary file/miRDeep2 file.zip!/miRDeep2 file/Know_Structure/SP42A3/oar-mir-26b.pdf]

miRBase precursor : oar-mir-26b
Total read count : 169871
oar-miR-26b read count : 169734
remaining reads : 137

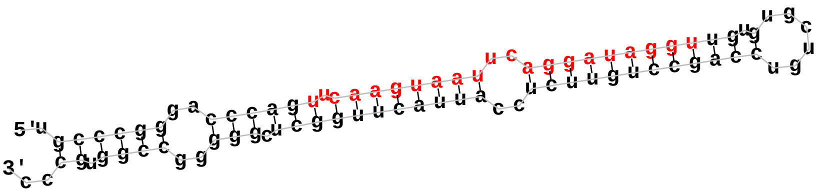

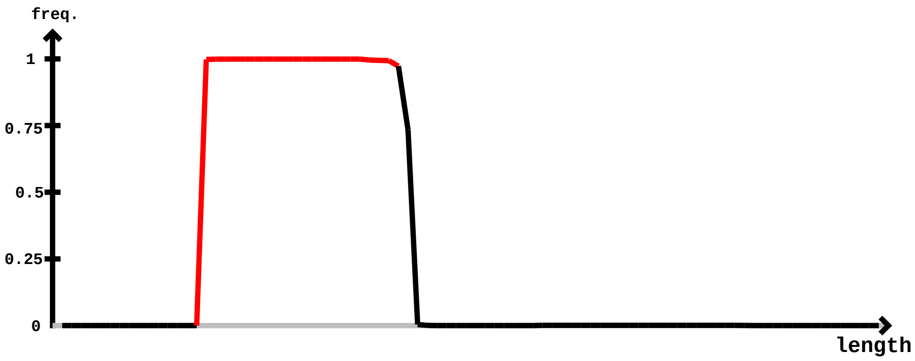

oar-miR-26b

5'- ugcccgggacccaguucaaguaauucaggauagguugugugcuguccagccuguucuccauuacuuggcucgggggccggugccc -3'	exp		
.((((((..((((((.((((((((..(((((((((((.(......))))))))))))..))))))))))).))).)))).))..	reads	mm	sample
.............guucaaguaauucaggauagguu...	1	0	seq
..............uucaaguaauucaggCu..	1	1	seq
..............uucaaguaauucaggaG..	8	1	seq
..............Aucaaguaauucaggau..	2	1	seq
..............uCcaaguaauucaggau..	1	1	seq
..............uucaaguaauucaggaC..	56	1	seq
..............uucaGguaauucaggau..	5	1	seq
..............uucaaguaauucagUau..	1	1	seq
..............uucCaguaauucaggau..	21	1	seq
..............uucaagCaauucaggau..	6	1	seq
..............uuUaaguaauucaggau..	1	1	seq
..............uucaaguaauucaggau..	497	0	seq
..............uucaaguaauucaggaA..	6	1	seq
..............uucaUguaauucaggau..	1	1	seq
..............uucaaguGauucaggau..	1	1	seq
..............uucaaguaauucaggaua...	211	0	seq
..............uucCaguaauucaggaua...	17	1	seq
..............uucaaguaauucaggaAa...	2	1	seq
..............uucaaguaauucaggauag..	122	0	seq
..............uucaaguaauucaggauagg...	3516	0	seq
..............uucaaguaauucaggauaggu..	40504	0	seq
..............uucaaguaauucaggauagguu...	123819	0	seq
..............uucaaguaauucaggauagguug..	603	0	seq
..............uucaaguaauucaggauagguugu...	88	0	seq
...............ucaaguaauucaggauag..	1	0	seq
...............ucaaguaauucaggauagg...	6	0	seq
...............ucaaguaauucaggauaggu..	40	0	seq
...............ucaaguaauucaggauagguu...	155	0	seq
...............ucaaguaauucaggauagguug..	2	0	seq
...............ucaaguaauucaggauagguugu...	2	0	seq
................caaguaauucaggauaggu..	4	0	seq
................caaguaauucaggauagguu...	21	0	seq
................caaguaauucaggauagguugu...	1	0	seq
.................aaguaauucaggauagg...	1	0	seq
.................aaguaauucaggauagguu...	10	0	seq

oar-miR-26b

```
ugcccgggacccaguucaaguaauucaggauagguugugugcuguccagccuguucuccauuacuuggcucgggggccggugccc
...................guaauucaggauagguu.................................................    1    0    seq
....................................gugugcuguccagccuguucuccauuacuuggcuc..............    3    0    seq
.......................................ugcuguccagccuguucuccauuacuuggcu...............    1    0    seq
.................................................ccuguucuccauuacuuggc................    2    0    seq
.................................................ccuguucuccauuacuuggcu...............    59   0    seq
.................................................ccuguucuccauuacuuggcuc..............    72   0    seq
```
